# Uptake of a new meningitis vaccination programme amongst first-year undergraduate students in the United Kingdom: A cross-sectional study

**DOI:** 10.1371/journal.pone.0181817

**Published:** 2017-08-02

**Authors:** Sarah Blagden, Daniel Seddon, Daniel Hungerford, Debbi Stanistreet

**Affiliations:** 1 Department of Public Health and Policy, Institute of Psychology Health and Society, University of Liverpool, Liverpool, United Kingdom; 2 NHS England, Regatta Place, Brunswick Business Park, Liverpool, United Kingdom; 3 The Centre for Global Vaccine Research, Institute of Infection and Global Health, University of Liverpool, Liverpool, United Kingdom; 4 Public Health England North West, Liverpool, United Kingdom; Dana-Farber Cancer Institute, UNITED STATES

## Abstract

**Background:**

In 2015 meningococcal group W was declared endemic in the UK, with the meningococcal ACWY vaccination (MenACWY) subsequently introduced amongst adolescents and first-year university students. This study aimed to determine MenACWY uptake amongst students and to evaluate how this was influenced by demographics and via the Health Belief Model (HBM).

**Methods:**

This was a cross-sectional study conducted at a British university amongst first-year undergraduate students aged 18–25 years. Data collection was via an electronic questionnaire encompassing demographics, the HBM and vaccination status. Univariable analysis of the associations between demographics, health beliefs and vaccination was performed, followed by multiple logistic regression.

**Results:**

401 participants were included in analysis. Vaccine uptake was 68.1%. Variables independently associated with vaccination upon multiple regression were age, gap-year, perceived effectiveness of the vaccine and knowledge about risk of meningitis. Compared to 18 year-olds, the odds of vaccination were reduced for 19 year-olds (aOR = 0.087, 95% CI = 0.010–0.729), 20 year-olds (aOR = 0.019, 95% CI = 0.002–0.161) and 21–25 year-olds (aOR = 0.003, 95% CI = <0.001–0.027). In contrast, taking a gap year (aOR = 2.939, 95% CI = 1.329–6.501), higher perceived vaccine effectiveness (aOR = 3.555, 95% CI = 1.787–7.073) and knowledge about meningitis risk (aOR = 2.481, 95% CI = 1.165–5.287) were independently associated with increased uptake.

**Conclusions:**

MenACWY uptake amongst students in this study and in other sources is above the national coverage for all adolescents (35.3%), indicating that this vaccination programme may be increasing health inequalities. Older students are less likely to become vaccinated due to differing vaccination policy in this age-group. In future, strategies that focus on specific student cohorts and that highlight vaccine effectiveness and the risk of meningitis should be considered. National evaluation of this vaccination programme is recommended to clarify its impact on health inequalities.

## Introduction

Since 2009 incidence of invasive meningococcal disease due to capsular group W (MenW) in the United Kingdom (UK) has been increasing [1,2]. Mortality has also increased, including, for the first time in the past decade, infant and adolescent deaths [2,3]. In early 2015, in light of accelerating incidence and mortality, MenW was declared endemic in the UK [1,2]. In response, the single-dose quadrivalent meningococcal ACWY (MenACWY) vaccine urgently replaced meningococcal C (MenC) vaccination in schools in 2015 and an urgent call/recall catch-up programme in primary care for children of school year 13 age (17–18 years-old) was implemented [3,4]. All students under 25 years of age attending university for the first time in Autumn 2015 were also recommended to become vaccinated [3].

This vaccination strategy reflects that adolescents and young adults have the highest carriage rates of meningococcal bacteria, with around 25% asymptomatically colonised [1,5]. Furthermore, young people, especially students, often live in close proximity in environments that facilitate bacterial transmission via droplet and aerosol routes [5]. These factors lead to this age-group experiencing the second highest rates of invasive meningococcal disease, after infants, in the population [5]. Furthermore, meningococcal carriage at this age drives transmission of MenW across the population, with the MenACWY vaccination programme anticipated to generate herd immunity [1].

In 2015, school-based vaccination of 13–15 year-olds began, whilst vaccination of students and of all young people aged 17–18 years was delivered in primary care [3,4]. Although UK students leaving school year 13 (aged 18 years) were amongst those actively invited for vaccination, this did not extend to older or international first-year students, where vaccination was limited to self-presenting individuals and opportunistic encounters [4].

As this is a new vaccination programme, there is very limited pre-existing research literature relating to its uptake. A recent study evaluated uptake of this vaccination programme at universities in Northern Ireland. Although it did not examine variables associated with vaccination, this study found that uptake varied from 87.3–90.7% amongst 18 year-old students and gradually fell with increasing age to 32.7–39.6% amongst 20–25 year old students [6]. In addition, another study evaluated uptake of the MenACWY vaccination at the University of Nottingham, UK following a mass campus vaccination campaign, which found that vaccine uptake was 71%, compared to 31% who had been vaccinated prior to arrival at university [7]. Crucially, however, this study identified that, even though all students were offered immediate free vaccination upon arrival at university, 43% of the unvaccinated population declined [7]. Thus, further work is essential to understand the factors that influence vaccination in this cohort. Four previous studies from the UK and United States (US) have also examined student uptake of meningococcal vaccines in the context of endemic MenC during the 1990s/early 2000s [8–11]. These reported vaccine uptake of between 51.0–87.0% and had examined several variables that affected vaccination, with factors such as age, university degree, gender and ethnicity significantly associated with vaccination in various studies [8–11].

The Health Belief Model (HBM) is a psychological behaviour change model which postulates that for somebody to access an intervention they must perceive that: they are susceptible to the disease, the disease is severe, the intervention would provide benefits and barriers would not be encountered [12,13]. Uptake may also be influenced by ‘cues to action’ and individual ‘self-efficacy’ [12,13]. Although not previously used within meningococcal vaccination, it has been widely used to understand student vaccination behaviour for other vaccinations such as influenza, measles and human papilloma virus (HPV) [14–23].

This study investigated MenACWY uptake amongst students at a UK university. As this is a new vaccination programme, research and evaluation are essential for several reasons. Firstly, although uptake data was collected by Public Health England (PHE) for children of school year 13 age, official estimates were complicated by the fact that students may receive vaccination at either their home or university GP practice. As there was no robust method for aligning these rates students registered at both their home and university GP practice may have appeared twice in the denominator. In addition, uptake data was not collected for older students. Therefore, this study will help to supplement official vaccination statistics. As discussed, this age-group has the highest meningococcal carriage rate and the second highest rate of invasive disease and it is essential to evaluate any attempt to address these public health concerns. This study is one of the first to evaluate this new vaccination programme and is the first to explore variables that are associated with uptake of this vaccination. This work contributes to wider evaluation of the vaccination programme and will help inform targeted MenACWY vaccination strategies, with the potential to also inform future strategies for other vaccines targeted at adolescents and young adults. Therefore, the study aimed to determine MenACWY uptake amongst first-year students and to ascertain how this was influenced by demographic characteristics and health beliefs. This aim was achieved.

## Material and methods

### Setting

This study was conducted at the University of Liverpool, UK. The university is situated in the centre of the city of Liverpool and serves a total population of 22,000 students, which included nearly 6,000 first-year undergraduate students in 2015/16 [24].

### Participants

Inclusion criteria were in accordance with the MenACWY vaccination service specification, with participants required to be a first-year undergraduate student, aged 18–25 years and attending university for the first time in Autumn 2015 [4].

### Study design and questionnaire

This was a community-based cross-sectional study that was conducted from March-May 2016 (questionnaire in S1 File). Data was collected via an electronic ‘SurveyMonkey’ questionnaire [25]. Throughout the survey, the term ‘meningitis’ was used instead of ‘invasive meningococcal disease’, for ease of reading and because it is a term that is more likely to be understood. The first item was a mandatory consent question that participants were required to accept in order to complete the survey summarising the study’s purpose, inclusion criteria, voluntary nature, confidentiality and right to withdraw. Confirmation was then sought that students were undertaking their first degree, and therefore met the eligibility criteria. Participants were next asked about their demographic characteristics, including their age, gender, ethnicity, university degree, home country and parental occupation (to determine socioeconomic group). Options for ethnicity were given in accordance with the 2015 Office for National Statistics (ONS) classification system for coding ethnicity in the UK [26].

Participants were subsequently asked yes/no items regarding experience of meningitis and MenACWY vaccination status. Participants were then directed to a HBM section depending on their vaccination status, as statements were phrased differently according to vaccination status. These items encompassed the following HBM domains: perceived severity, susceptibility, barriers, benefits and cues to action. Answers were given on a five-point Likert scale from 1 (strongly disagree) to 5 (strongly agree). This section was based upon a previously validated instrument from a US study of pandemic influenza vaccination by Coe et al (2012) [17]. This tool was chosen because information was provided regarding its development and constituent items and because influenza, like meningococcal disease, is an acute infectious disease spread by droplet transmission that has experienced recent resurgences [17,27–29].

Some adjustments were made to the survey instrument. A question on vaccine cost was removed to reflect the free provision of UK healthcare and items pertaining to knowledge of meningitis and the impact of family and friends were incorporated to reflect evidence that these factors have previously influenced vaccination uptake [10,17,30,31]. In light of these amendments, further validation was undertaken. Twenty individuals piloted the questionnaire, with two items removed following this process. These related to loss of income following illness, which was considered irrelevant to most students and risk of death associated with vaccination, which was thought to pose potential anxieties and be inappropriate for an established vaccine. Cronbach’s alpha was then calculated for each HBM domain to evaluate the reliability and internal consistency of the instrument, with good internal consistency for most domains (Cronbach’s alpha = 0.494–0.813) (Table 1).

**Table 1:** Results of Cronbach’s alpha for Health Belief Model (HBM) survey domains.

HBM Domain	Cronbach’s alpha
Perceived severity of meningitis	0.494
Perceived susceptibility to meningitis	0.701
Barriers to vaccination	0.745
Benefits of vaccination	0.580
Cues to action	0.813

Upon survey completion students were directed to a leaflet that contained information about meningitis, the vaccine, the study and additional resources and contact details.

### Sampling

Participants were recruited via email through University of Liverpool academic departments, followed by a reminder email two weeks later. All university departments with first-year undergraduate students enrolled were approached regarding study participation and those that agreed forwarded the survey via email to the first-year students in their department, encompassing around 3,000 students. A wide spectrum of degree programmes participated in the study.

### Ethics

This project received ethical approval from the University of Liverpool Institute of Psychology Health Society Ethics Committee (approval number: IPHS-1516-LB-189). Informed valid consent was gained from study participants via the consent question at the beginning of the electronic questionnaire.

### Analysis

All analyses were conducted using IBM SPSS statistics version 22. The first stage of analysis was data management. Where participants had only provided consent and/or demographic responses or had indicated they were not undertaking their first degree, they were excluded. Following this, available case analysis incorporating all participants who had responded to the variable(s) of interest was conducted. Several variables were recoded, with parental occupations recoded into the National Statistics Socio-economic Classification (NS-SEC) coding tool that is used to assign socio-economic analytical class based upon occupation in the UK [32]. Here, groups were further categorised into groups 1–4 and 5–8, which are often used to differentiate between advantaged and disadvantaged groups [32,33]. Undergraduate degrees were recoded into larger groups and, due to small counts for some ethnic minorities, ethnicity was recoded into “White British” and “Other ethnic groups”. In order to make HBM responses more meaningful, Likert scale responses 1–3 were re-categorised as “Disagree/neither agree nor disagree” and responses 4–5 as “Agree”. Age was treated as a categorical variable and sub-divided into four categories: 18 years, 19 years, 20 years and 21–25 years. This grouping of older students was used because in the UK students aged 21 years and above are classified as “mature students” [34].

For the sample overall, and for vaccinated and unvaccinated cohorts, summary statistics were calculated and tabulated. Univariable logistic regression was then undertaken to explore the association between independent variables (demographics and health beliefs) and the dependent variable (vaccination). Univariable associations were statistically significant if p≤0.05.

Finally, multiple logistic regression was used to investigate the effect of independent variables on vaccination, adjusted for the effect of confounders. Variables were entered into the multivariable model if, upon univariable regression analysis, the p value was ≤0.2. In addition, gender, age and ethnicity were entered into the model a priori. The goodness of fit and predictive properties of the multivariable model were assessed using the Chi-square value and the Nagelkerke R square value.

## Results

### Response rate

In total, 485 individuals responded to the survey, of which 57 (11.8%) had answered the consent statement only and a further 27 (5.6%) had provided either solely demographic responses and/or had indicated that this was not their first degree. These individuals were excluded. Therefore, the final sample contained 401 participants. As approximately 3,000 students had been forwarded the survey, this represents a crude response rate of 13.4%.

### Vaccination uptake

Self-reported vaccination uptake was 68.1%, with 273 participants reporting that they had received the MenACWY vaccination and 128 reporting that they had not.

### Sample characteristics

The median age of participants was 20 years and the majority were female (78.1%) (Table 2). The sample was mainly comprised of UK students (93.5%) and 28.9% of participants had taken a gap-year. Overall, 80% of the sample were White British. The sample was skewed towards more advantaged socio-economic groups (81.3%).

**Table 2:** Demographic characteristics of study participants and results of univariable regression analysis of associations between demographic variables and vaccination.

Categorical variable	All students	Vaccinated students	Unvaccinated students	Odds ratio (95% confidence interval)	P value
	N (%)*				
**Age (years):**					
18	71 (17.7)	69 (25.3)	2 (1.6)	1	
19	120 (29.9)	107 (39.2)	13 (10.2)	0.239 (0.052–1.090)	0.064
20	89 (22.1)	58 (21.2)	31 (24.2)	0.054 (0.012–0.236)	<0.001
21–25	113 (28.2)	36 (13.2)	77 (60.2)	0.014 (0.003–0.058)	<0.001
**Gender:**					
Males	85 (21.2)	63 (23.0)	22 (17.2)	1	
Females	313 (78.1)	208 (76.2)	106 (82.8)	0.692 (0.400–1.174)	0.18
**Country of origin:**					
Home (UK) students	375 (93.5)	266 (97.4)	109 (85.2)	1	
International students	26 (6.5)	7 (2.6)	20 (15.6)	0.151 (0.062–0.369)	<0.001
**Gap-year status:**					
Gap-year not taken	285 (71.1)	202 (74.0)	83 (64.8)	1	
Gap-year taken	116 (28.9)	71 (26.0)	46 (35.9)	0.648 (0.412–1.019)	0.06
**Ethnicity:**					
White British	321 (80.0)	225 (82.4)	96 (75.0)	1	
Any other ethnic group	76 (19.0)	48 (17.6)	28 (21.9)	0.857 (0.071–10.331)	0.903
**Socio-economic group based on grouping of National Statistics Socio-economic classification (NS-SEC) tool:**					
More advantaged groups (NS-SEC groups 1–4)	326 (81.3)	223 (81.7)	103 (80.5)	1	
Less advantaged groups (NS-SEC groups 5–8)	54 (13.5)	40 (14.7)	14 (10.9)	0.758 (0.395–1.454)	0.404
**University degrees:**					
Medicine	45 (11.2)	42 (15.4)	3 (2.3)	1	
Art	47 (11.7)	29 (10.6)	18 (14.1)	0.115 (0.031–0.427)	0.001
Other vocational Health Science	50 (12.5)	35 (12.8)	15 (11.7)	0.167 (0.045–0.623)	0.008
Maths and Science degrees	115 (28.7)	73 (26.7)	42 (32.8)	0.124 (0.036–0.425)	0.001
Geography	25 (6.2)	23 (8.4)	2 (1.6)	0.821 (0.128–5.277)	0.836
Psychology	64 (16.0)	35 (12.8)	29 (22.7)	0.086 (0.024–0.307)	<0.001
Engineering or Architecture	34 (8.5)	27 (9.9)	7 (5.5)	0.276 (0.066–1.159)	0.079
**Personal or close (friend or family member) experience of meningitis:**					
Yes	52 (13.0)	38 (13.9)	14 (10.9)	1	
No	348 (87.0)	235 (86.1)	113 (88.3)	1.305 (0.680–2.506)	0.424

*Not all totals add up to n = 401 (100%) due to missing data for some variables.

When considering health beliefs across the sample (Table 3), 89.3% of participants perceived that meningitis would cause severe disease if they were to become infected, although only 20.4% believed that it would cause death. In terms of susceptibility, although 69.6% believed they were at risk of getting meningitis, only 26.7% felt knowledgeable about their risk of meningitis. There was low perception of barriers to vaccination. In contrast, there was high perception of the general benefits of vaccination, with 75.3% believing vaccines prevent disease and 73.3% believing them to be safe. However, when considering the MenACWY vaccination specifically, only 55.4% perceived that it prevented meningitis. Doctor recommendation was a widely perceived cue to action, with 82.3% of participants believing themselves more likely to accept a vaccination if recommended by a doctor.

**Table 3:** Health beliefs of study participants and results of univariable regression analysis of associations between demographic variables and vaccination.

Independent variable	All students	Vaccinated students	Unvaccinated students	Odds Ratio (95% confidence interval)	P value
	N (%)*				
**Perceived severity of meningitis**					
Likelihood of severe personal illness if infected with meningitis:					
Disagree/neither agree nor disagree	42 (10.5)	22 (8.1)	20 (15.6)	1	
Agree	358 (89.3)	250 (91.6)	108 (84.4)	2.104 (1.103–4.016)	0.024
Likelihood of onward transmission to family/friends if infected with meningitis:					
Disagree/neither agree nor disagree	156 (38.9)	102 (37.4)	54 (42.2)	1	
Agree	242 (60.3)	168 (61.5)	74 (57.8)	1.202 (0.783–1.845)	0.4
Likelihood of death if infected with meningitis:					
Disagree/neither agree nor disagree	314 (78.3)	213 (78.0)	101 (78.1)	1	
Agree	82 (20.4)	57 (20.9)	25 (19.5)	1.081 (0.639–1.830)	0.771
**Perceived susceptibility to meningitis**					
At personal risk of getting meningitis:					
Disagree/neither agree nor disagree	117 (29.2)	192 (70.3)	87 (68.)	1	
Agree	279 (69.6)	78 (28.6)	38 (29.7)	0.906 (0.572–1.436)	0.906
Family and friends at risk of getting meningitis:					
Disagree/neither agree nor disagree	297 (74.1)	199 (72.9)	98 (76.6)	1	
Agree	98 (24.4)	70 (25.6)	28 (21.9)	1.231 (0.746–2.031)	0.416
Knowledgeable about risk of getting meningitis:					
Disagree/neither agree nor disagree	291 (72.6)	186 (68.1)	105 (82.0)	1	
Agree	107 (26.7)	85 (31.1)	22 (17.2)	2.181 (1.288–3.692)	0.004
Knowledgeable about meningitis in general:					
Disagree/neither agree nor disagree	296 (73.8)	201 (73.6)	95 (74.2)	1	
Agree	101 (25.2)	70 (25.6)	31 (24.2)	1.067 (0.655–1.739)	0.794
**Barriers to vaccination**					
Side effects associated with MenACWY vaccination:					
Disagree/neither agree nor disagree	337 (84.0)	228 (83.5)	109 (85.2)	1	
Agree	47 (11.7)	36 (13.2)	11 (8.6)	1.565 (0.767–3.191)	0.218
Illness associated with MenACWY vaccination:					
Disagree/neither agree nor disagree	370 (92.3)	253 (92.7)	117 (91.4)	1	
Agree	15 (3.7)	12 (4.4)	3 (2.3)	1.850 (0.512–6.680)	0.348
Pain associated with MenACWY vaccination:					
Disagree/neither agree nor disagree	321 (80.1)	217 (79.5)	104 (81.3)	1	
Agree	63 (15.7)	47 (17.2)	16 (12.5)	1.408 (0.762–2.600)	0.275
Inconvenience associated with MenACWY vaccination:					
Disagree/neither agree nor disagree	357 (89.1)	252 (92.3)	105 (82.0)	1	
Agree	29 (7.2)	14 (5.1)	15 (11.7)	0.389	0.015
Perceived shortage of MenACWY vaccination:					
Disagree/neither agree nor disagree	350 (87.3)	247 (90.5)	103 (80.5)	1	
Agree	33 (7.2)	18 (6.6)	15 (11.7)	0.500 (0.243–1.031)	0.06
**Benefits of vaccination**					
The MenACWY vaccination is effective at preventing meningitis:					
Disagree/neither agree nor disagree	163 (40.5)	88 (32.2)	75 (58.6)	1	
Agree	222 (55.4)	177 (64.8)	45 (35.2)	3.352 (2.139–5.254)	<0.001
Vaccines prevent disease:					
Disagree/neither agree nor disagree	79 (19.7)	52 (19.0)	27 (21.1)	1	
Agree	302 (75.3)	211 (77.3)	91 (71.1)	1.204 (0.712–2.037)	0.489
Vaccines are safe:					
Disagree/neither agree nor disagree	87 (21.7)	52 (19.0)	27 (21.1)	1	
Agree	294 (73.3)	211 (77.3)	91 (71.1)	1.711 (1.040–2.816)	0.035
**Cues to action**					
Likely to accept vaccination if recommended by a doctor:					
Disagree/neither agree nor disagree	49 (12.2)	31 (11.4)	18 (14.1)	1	
Agree	330 (82.3)	231 (84.6)	99 (77.3)	1.355 (0.724–2.536)	0.342
Likely to accept vaccination if recommended by a pharmacist:					
Disagree/neither agree nor disagree	164 (40.9)	104 (38.1)	60 (46.9)	1	
Agree	215 (53.6)	157 (57.5)	58 (45.3)	1.562 (1.008–2.419)	0.046
Likely to accept vaccination if recommended by a nurse:					
Disagree/neither agree nor disagree	103 (25.7)	63 (23.1)	40 (31.3)	1	
Agree	277 (69.1)	199 (72.9)	78 (60.9)	1.620 (1.007–2.605)	0.047
Likely to accept vaccination if recommended by family or friends:					
Disagree/neither agree nor disagree	221 (55.2)	143 (52.4)	78 (60.9)	1	
Agree	158 (39.4)	118 (43.2)	40 (31.3)	1.609 (1.023–2.530)	0.039
Likely to accept vaccination based on the behaviour of peers:					
Disagree/neither agree nor disagree	217 (54.2)	150 (54.9)	67 (52.3)	1	
Agree	163 (40.6)	112 (41.0)	51 (39.8)	0.932 (0.632–1.521)	0.931

*Not all totals add up to n = 401 (100%) due to missing data for some variables.

### Univariable analysis

Age, international student status and university degree were associated with vaccination uptake (Table 2). Age was negatively associated with vaccination uptake, with the odds of vaccination reducing with increasing age. Similarly, international students were significantly less likely to become vaccinated. Compared to the degree cohort with the highest uptake, Medicine, students undertaking Arts, other vocational Health Sciences, Maths and Sciences and Psychology degrees had significantly reduced odds of vaccination.

Looking at the HBM (Table 3), students who believed meningitis would cause severe illness, who perceived themselves to be knowledgeable about their meningitis risk and who perceived that the MenACWY vaccine effectively prevents meningitis were more likely to become vaccinated. Students who believed vaccines to be safe were also more likely to become vaccinated, along with participants who were more likely to accept a vaccination if recommended by a pharmacist, nurse or friend/family member. In contrast, students who perceived that receiving MenACWY vaccination was inconvenient had reduced odds of vaccination.

### Multivariable analysis

The multivariable model generated a large Chi-square statistic (170.340) and predicted vaccination significantly well (p<0.001). The Nagelkerke R square value of 0.556 demonstrated that 55.6% of the variability in vaccination was predicted by the multivariable model. Age was independently associated with vaccination, with, when compared to 18 year-olds, reduced odds of vaccination amongst 19 year-olds (adjusted odds ratio (aOR) = 0.087, 95% confidence interval (CI) = 0.010–0.729), 20 year-olds (aOR = 0.019, 95% CI = 0.002–0.161) and 21–25 year-olds (aOR = 0.003, 95% CI = <0.001–0.027). The other variables independently associated with vaccination were taking a gap-year (aOR = 2.939, 95% CI = 1.329–6.501), perceived knowledge about risk of meningitis (aOR = 2.481, 95% CI = 1.165–5.287) and perceived vaccine effectiveness (aOR = 3.555, 95% CI = 1.787–7.073) (Table 4).

**Table 4:** Multivariable logistic regression analysis of the associations between health beliefs, demographic variables and vaccination.

Independent variable	Adjusted odds ratio (95% confidence interval)	P value
**Age (years):**		
18	1	
19	0.087 (0.010–0.729)	0.024
20	0.019 (0.002–0.161)	<0.001
21–25	0.003 (<0.001–0.027)	<0.001
**Gender:**		
Males	1	
Females	0.725 (0.260–2.024)	0.725
**Ethnicity:**		
White British	1	
Any other ethnic group	0.920 (0.303–2.799)	0.771
**Country of origin:**		
Home (UK) students	1	
International students	0.968 (0.147–6.369)	0.884
**University degrees:**		
Medicine	1	
Arts	0.335 (0.048–2.324)	0.269
Other vocational Health Science	0.318 (0.047–2.164)	0.242
Maths and Science	0.454 (0.077–2.664)	0.382
Geography	0.553 (0.043–7.194)	0.651
Psychology	0.161 (0.025–1.022)	0.053
Engineering and Architecture	0.383 (0.046–3.216)	0.376
**Gap-year status:**		
Gap-year not taken	1	
Gap-year taken	2.939 (1.329–6.501)	0.008
**Severe illness associated with vaccination:**		
Disagree/neither agree nor disagree	1	
Agree	0.821 (0.260–2.592)	0.736
**Knowledgeable about risk of meningitis:**		
Disagree/neither agree nor disagree	1	
Agree	2.481 (1.165–5.287)	0.019
**Inconvenience associated with MenACWY vaccination:**		
Disagree/neither agree nor disagree	1	
Agree	0.480 (0.140–1.643)	0.242
**Perceived shortage of MenACWY vaccine:**		
Disagree/neither agree nor disagree	1	
Agree	0.518 (0.158–1.696)	0.277
**The MenACWY vaccination is effective at preventing meningitis:**		
Disagree/neither agree nor disagree	1	
Agree	3.555 (1.787–7.073)	<0.001
**Vaccines are safe:**		
Disagree/neither agree nor disagree	1	
Agree	1.951 (0.858–4.438)	0.111
**Likely to accept vaccination if recommended by a pharmacist:**		
Disagree/neither agree nor disagree	1	
Agree	1.048 (0.448–2.452)	0.915
**Likely to accept vaccination if recommended by a nurse:**		
Disagree/neither agree nor disagree	1	
Agree	0.919 (0.363–2.325)	0.858
**Likely to accept vaccination if recommended by friends or family:**		
Disagree/neither agree nor disagree	1	
Agree	1.533 (0.756–3.105)	0.236

## Discussion

This is one of the first studies to evaluate the MenACWY vaccination programme in the UK and the first to explore variables associated with vaccination. We found that amongst study participants, MenACWY uptake was 68.1%. Data on vaccine uptake had not been routinely collected by the University of Liverpool, however in order to assess this figure’s representativeness, other data sources were examined. Amongst the school year 13 age-group that were actively called for vaccination, the national coverage reported by PHE was 35.2% [35]. Data provided by NHS England Cheshire and Merseyside of the Liverpool GP practices with large numbers of university students and data from the Student Wellbeing Department at Liverpool Hope University, another university in Liverpool, demonstrated vaccine uptake of between 40–68.6% [36]. The recent study by Moore et al in Northern Irish universities reported an uptake of between 32.7% and 90.7% depending on the age and sex of the students [6]. However, this study included some non-first time students who would not be eligible for vaccination and the uptake figures for older students may have underestimated uptake [6]. Turner et al reported 71% uptake of the MenACWY vaccination amongst first-years at the University of Nottingham following a campus-based mass vaccination campaign and the four previous studies of student meningococcal vaccination from the 1990s/2000s reported uptake of 51–87% [7–11].

Thus, although our study looked at a single university and is by no means representative of all UK students, it seems likely that student MenACWY vaccine uptake was closer to the figure reported in this study, above the national uptake and, therefore, above that of non-student adolescents. This may reflect the educational attainment of students as well as efforts by universities, GPs and organisations such as PHE to vaccinate students. For example, although the university did not routinely check the vaccination status of all new arrivals, the MenACWY vaccination was advertised heavily during “Welcome Week” at the University of Liverpool and drop-in vaccination sessions were provided on campus by the local GP practice to allow opportunistic vaccination of students. This is in line with the findings of Turner et al who demonstrated that offering vaccination upon immediate arrival at university increased uptake from 31% of students who had been vaccinated by their GP prior to arrival at university to 71% [7]. This higher student uptake, therefore, provides support for these efforts. Although students are at slightly increased risk of meningococcal disease due to living in close proximity to each other in environments such as halls of residence, non-students are more likely to come from disadvantaged backgrounds and live in deprived areas [37]. There is a considerable body of evidence suggesting that deprivation is linked to increased incidence of invasive meningococcal disease in the UK due to factors such as overcrowded living conditions and increased rates of smoking, including passive smoking, that increase the likelihood of both asymptomatic meningococcal carriage and invasive disease [38–42]. Thus, it is concerning that non-students do not have access to the efforts aimed at students and are likely to have experienced far lower vaccination uptake.

This study found that vaccination rates decreased with increasing age and has provided a unique insight into vaccination amongst 20–25 year-olds, with data on uptake in this age-group not collected by PHE [35]. This finding concurs with other studies of student meningococcal vaccination and is highly relevant given that many students enter university at an older age, with 50.4% of the sample aged 20–25 years [6,8,9]. As this association was amplified upon multivariable analysis, it is unlikely to be attributable to demographics or health beliefs. The key difference, therefore, between younger and older students was the difference in vaccination policy, with vaccination opportunistic amongst older students instead of active call/recall [4]. For GP practices and universities, this represents a focus for future vaccination efforts as younger students will increasingly receive vaccination in schools [3]. As with the likely lower vaccination uptake amongst non-students, further evaluation is needed to confirm this inequality in vaccination uptake and, if confirmed, to establish why this exists in order to prevent future vaccination programmes from perpetuating health inequalities.

An interesting finding was the positive association between vaccination and gap-year, despite univariable analysis suggesting a negative relationship. However, the fact that this was a self-reported variable and relied upon older students self-identifying as having taken a gap-year may have introduced confounding. An alternative explanation is that many students use their gap-year to travel to developing countries where MenACWY is a recommended travel vaccination [43].

Looking at the HBM, this study found that perceived effectiveness of MenACWY was significantly associated with uptake. This concurs with previous literature suggesting that a vaccine’s perceived ability to prevent disease is commonly the most important vaccination determinant [15,16,22,44–46]. Although in the minority in vaccinated and unvaccinated cohorts, students who perceived themselves as knowledgeable about their meningitis risk were significantly more likely to accept vaccination than those who did not. This finding supplements previous studies, whereby misconceptions about influenza, measles and HPV susceptibility were associated with vaccine rejection [15,20,44,46].

These findings represent potential focus areas in future vaccination campaigns. Such campaigns could target specific student groups, such as older students, and should include information on vaccine effectiveness and the risk of meningitis amongst students. As the literature has demonstrated that such factors may be relevant to other vaccinations, these findings could potentially inform strategies for other vaccinations in this age-group.

### Strengths and limitations

This study was subject to several limitations inherent to cross-sectional studies. Firstly, the response rate of 13.4% indicates that response bias may have occurred, although this is likely to have been an underestimate due to the denominator used inevitably including students not eligible for vaccination; for example, those over 25 years-old. Furthermore, uptake may have been underestimated due to factors such as participants not being aware of what they were vaccinated against [31,47].

Demographic data obtained from the University of Liverpool demonstrated that 55% of first-year undergraduates in 2015/16 were female and 76.3% were White [48]. Thus, although the proportion of White students was fairly representative (80%), proportionally more females (78.1%) participated in the study. However, as we do not have the breakdown of each degree programme by gender, it is not possible to determine whether this female predominance was due to a response bias and an increased willingness of females to complete the survey or female dominance within participating departments.

The use of convenience sampling represents a further drawback and may have resulted in selection bias, with certain academic departments not participating in the study. Like response bias, this limits the generalisability of findings. Linked to this, the University of Liverpool is a highly prestigious university in the UK and its students may not be representative of the general population. It is noted, however, that similar uptake figures were obtained in the recent papers from Northern Ireland by Moore et al and the paper from the University of Nottingham by Turner et al, which increases the likelihood that the results from this study are representative of students and generalizable to the wider student population in the UK [6,7]. Although statistical techniques were used to adjust for confounding, some confounding introduced by response bias, selection bias and the sample taken from a single university is likely to remain. Whilst significant associations were identified, causality cannot be inferred and the mechanism behind associations may be unclear [49].

Although the HBM is a useful and widely accepted model by which to structure beliefs that influence vaccination, it is not all encompassing and it is probable that there will have been other predictors of vaccine uptake that were not examined in this study; for example, the HBM assumes that students are already aware of the vaccination programme, which may not be the case. Similarly, demographic factors that were not examined in this study, such as whether students lived in halls of residence or not, may also have influenced vaccination uptake. A further critique of the HBM is that, whilst it is able to identify factors associated with vaccination, it is not possible to determine the relative importance of each of these factors in influencing vaccination.

In terms of strengths, this is one of the first studies to evaluate the MenACWY vaccination programme in the UK and the first to comprehensively evaluate variables associated with its uptake via multivariable analysis and a validated framework such as the HBM. The dependent variable was actual vaccination instead of intended uptake, which, with intentions not always translating into behaviour, is more meaningful when addressing behaviour. Furthermore, unlike other studies, data collection was undertaken when the year’s vaccination programme was complete to allow accurate determination of uptake. A large number of students participated, providing a considerable number of individuals at high-risk of meningitis due to their age and student status. The use of multivariable regression was a key strength as it allowed adjustment for confounders, with similar studies not utilising this technique [6,7,10,11]. The use of regression analysis enabled variables to be identified that were significantly associated with MenACWY vaccination in the UK, with the potential to inform future targeted vaccination strategies.

## Conclusions

This study’s findings suggest that there may be inequalities in MenACWY vaccine uptake, which warrant further evaluation and research, with potential implications for future policy if confirmed. Younger students and gap-year students were more likely to receive vaccination. Vaccination uptake was also affected by health beliefs, with students more likely to become vaccinated if they perceived the vaccine to be effective and if they were knowledgeable about their risk of meningitis. These health beliefs and demographic associations should be considered in future vaccination campaigns and additional research is recommended to explore them further.

## Supporting information

S1 File Final participant questionnaire. (DOCX)
