# Supplementary material for: Uptake of a new meningitis vaccination programme amongst first-year undergraduate students in the United Kingdom: A cross-sectional study
Source: PLoS One. 2017 Aug 2;12(8):e0181817. doi: 10.1371/journal.pone.0181817 (PMC5540503; doi:10.1371/journal.pone.0181817)
Supplement: S1 File — (DOCX) [file pone.0181817.s001.docx]

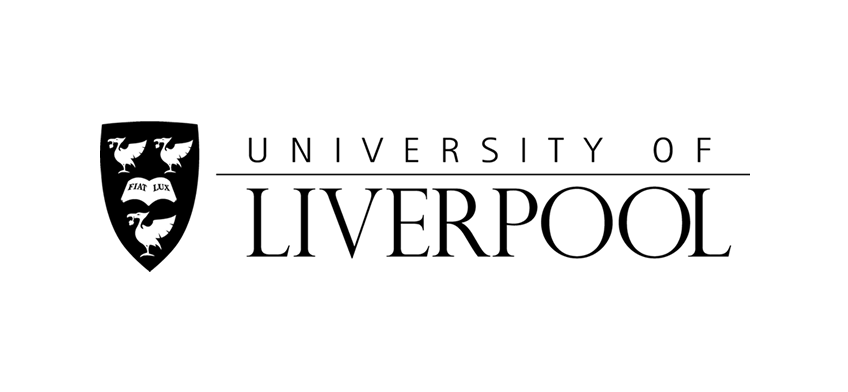
**Final Participant Questionnaire**

This survey is about meningitis and the vaccination against meningitis that was introduced in 2015 in first-year undergraduate students under the age of 25 years.

The survey is part of a research project that aims to explore the uptake of this vaccine, and the factors associated with this, in first-year undergraduates at the University of Liverpool. The data provided will help future strategies to maximise uptake of this vaccine.

All your responses to the survey are confidential and no data will be collected that could identify you in any way. Participation is completely voluntary and you are free to stop and exit the survey at any time during completion if you do not wish to continue for any reason. However, as the survey is confidential, once you submit your responses they cannot be withdrawn from the research project as it will not be possible to identify which responses were yours.

Upon completion of the survey, you will be provided with an information leaflet about meningitis and the vaccine.

If you have already completed this questionnaire please exit the survey. If you are older than 25 years or not attending university for the first-time in 2015 please exit the survey.

Please tick/click the box below to indicate your consent to the above

(mandatory item)

**Demographics**

- How old are you? (Drop-down box with options age 18-25years)
- Are you male or female? (Drop-down box with options “male” or “female”)
- Which university degree are you doing? (Free-typing)
- Is this your first university degree? (Drop-down box with options “Yes” or “No”)
- Are you an international student? (Drop-down box with options “Yes” or “No”)
- What is your home country? Question only triggered if answered “Yes” to preceding question (Free-typing)
- Did you take a gap-year? (Drop-down box with options “Yes” or “No”)
- What is your ethnicity? (Drop-down box with the following options)

**White:**

British/English/Welsh/Scottish/Northern Irish

Irish

Gypsy or Irish Travellers

Any other white background (please describe)………………………………

**Mixed/multiple ethnic groups**

White and Black Caribbean

White and Black African

White and Asian

Any other mixed ethnic background (please describe)………………………….

**Asian British/Asian**

Indian

Pakistani

Bangladeshi

Chinese

Any other Asian background (please describe)…………………………

**Black British/Black/African/Caribbean**

African

Caribbean

Any other Black/African/Caribbean background (please describe)…………………

**Other ethnic group**

Arab

Any other ethnic group (please describe)……………………………..

- Please state the occupation of one of your parents or guardians (Free-typing)

**Meningitis ACWY vaccine**

The meningitis ACWY vaccine is the vaccine against four strains of meningitis that was introduced in 2015 amongst school children and first-year undergraduate students. You may have received it over summer 2015 at home or in Liverpool upon arrival at university.

- Have you received the meningitis ACWY vaccine? (Mandatory item - drop-down box with options “Yes” or “No”)
- Have you or a close friend or family member of yours ever had meningitis? (Drop-down box with options “Yes” or “No”)

**The Health Belief Model**

**Please rate the extent to which you agree with the following statements, from 1 (don’t agree at all) to 5 (agree completely).**

(For all subsequent items responses provided using drop-down boxes with answers 1-5)

**For all participants:**

- If I get meningitis I will get very ill.
- If I get meningitis other family members or friends could get ill.
- If I get meningitis I will die.
- I am at risk of getting meningitis.
- My family and friends are at risk of getting meningitis.
- I feel knowledgeable about my risk of getting meningitis.
- I feel knowledgeable about meningitis in general.

(Skip question function then used to direct participants to the appropriate section depending on vaccination status)

**For unvaccinated participants:**

- If I have the MenACWY vaccine I will have side effects from it.
- If I have the MenACWY vaccine I will get ill from having it.
- If I have the MenACWY vaccine it will be painful.
- It is inconvenient to get the meningitis ACWY vaccine.
- There is a shortage of the meningitis ACWY vaccine.
- If I receive the meningitis ACWY vaccine, I will not get ill with meningitis.

**For vaccinated participants:**

- Before I had the meningitis ACWY vaccine, I thought I would have side effects from the vaccine.
- Before I had the meningitis ACWY vaccine, I thought I would get ill from having the vaccine.
- Before I had meningitis ACWY vaccine, I thought it would be painful.
- Before I had the meningitis ACWY vaccine, I thought it would be inconvenient to get the vaccine.
- Before I had the meningitis ACWY vaccine, I thought there was a shortage of the vaccine.
- I think having the meningitis ACWY vaccine will prevent me from getting ill with meningitis.

**For all participants:**

- Vaccines prevent disease.
- Vaccines are safe.
- I am more likely to have a vaccine if it is recommended by a doctor.
- I am more likely to have a vaccine if it is recommended by a pharmacist.
- I am more likely to have a vaccine if it is recommended by a nurse.
- I am more likely to have a vaccine if it is recommended by a family member of friend.
- I am more likely to have a vaccine if my friends have had it.

Thank you very much for taking part in this survey. You will now be directed to an information leaflet about meningitis and the meningitis ACWY vaccine, including information about how to become vaccinated if you have not already done so.
